# Training for child and adolescent psychiatry in the twenty-first century

**DOI:** 10.1007/s00787-019-01467-6

**Published:** 2020-01-16

**Authors:** Peter Deschamps, Johannes Hebebrand, Brian Jacobs, Paul Robertson, Dimitris C. Anagnostopoulos, Tobias Banaschewski, Sarah M. Birkle, Bernadka Dubicka, Bruno Falissard, Ioanna Giannopoulou, Pieter J. Hoekstra, Michael Kaess, Krisztina Kapornai, Paul Klauser, Alexis Revet, Carmen M. Schröder, Jochen Seitz, Asilay Şeker, Giulia Signorini

**Affiliations:** 1 Department of Psychiatry, University Medical Centre Utrecht, Utrecht, The Netherlands; 2 Department of Child and Adolescent Psychiatry, Psychosomatics and Psychotherapy, University Hospital Essen (AöR), Wickenburgstr. 21, 45147 Essen, Germany; 3 Child and Adolescent Psychiatry, South London and Maudsley Hospital, London, UK; 4 Department of Psychiatry, University of Melbourne, Melbourne, VIC Australia; 5 Medical School, National and Kapodistrian University of Athens, Athens, Greece; 6 Department of Child and Adolescent Psychiatry, Medical Faculty Mannheim, Central Institute of Mental Health, University of Heidelberg, Heidelberg, Germany; 7 Department of Psychosomatic Medicine and Psychotherapy, University Hospital Tuebingen, Tuebingen, Germany; 8 University of Manchester, Manchester, UK; 9 Centre de Recherche en Epidemiologie et Santé des Populations, Paris, France; 10 Department of Psychiatry, Medical School, National and Kapodistrian University of Athens, Attikon University Hospital, Athens, Greece; 11 Department of Child and Adolescent Psychiatry, University of Groningen, University Medical Center Groningen, Groningen, The Netherlands; 12 University Hospital of Child and Adolescent Psychiatry and Psychotherapy, University of Bern, Bern, Switzerland; 13 Department of Child and Adolescent Psychiatry, Center for Psychosocial Medicine, University Hospital Heidelberg, Heidelberg, Germany; 14 Child and Adolescent Psychiatry Department, University of Szeged, Szeged, Hungary; 15 Service of Child and Adolescent Psychiatry, Department of Psychiatry, Lausanne University Hospital, Lausanne, Switzerland; 16 Service Universitaire de Psychiatrie de l’Enfant et de l’Adolescent, CHU de Toulouse, Toulouse, France; 17 UMR 1027, Inserm, Université Toulouse III, Toulouse, France; 18 Department of Child and Adolescent Psychiatry, Strasbourg University Hospital, Strasbourg, France; 19 Institute of Cellular and Integrative Neurosciences, CNRS UPR 3212, Strasbourg, France; 20 Department of Child and Adolescent Psychiatry, Psychotherapy and Psychosomatics, University Hospital, RWTH University Aachen, Aachen, Germany; 21 Child and Adolescent Psychiatry Department, Erciyes University Hospital, Kayseri, Turkey; 22 European Federation of Psychiatric Trainees, Brussels, Belgium; 23 IRCCS Istituto Centro San Giovanni di Dio Fatebenefratelli, Brescia, Italy; 24 Pennine Care Foundation Trust, Ashton-under-Lyne, UK; 25 Section of Child and Adolescent Psychiatry, European Union of Medical Specialists (UEMS-CAP), Brussels, Belgium

We are a relatively young medical specialty and have found our roots in the past century in a combination of medicine (adult psychiatry and pediatrics), psychology and cooperation with various professionals involved with child/youth care and education. These historical origins are reflected in our activities today and we have witnessed a growth in number of both child and adolescent psychiatrists and trainees over the past decades. At the same time, there has also been a tremendous increase in demand for child and adolescent mental health services. It is becoming very clear that the sheer numbers of children and young people with impairing and diagnosable mental health difficulties means that it is likely to be a Herculean task to train enough high-quality mental health specialists/therapists. Some complex cases can wait several years before being properly understood diagnostically and getting appropriate help. This delay is distressing for the young patients and their families, contributes to chronicity and misses the opportunity for early intervention as well as wastes resources that could be more effectively used.

This special issue of *European Child and Adolescent Psychiatry* titled “The European and global perspective on training in child and adolescent psychiatry” takes an updated global look at training in child and adolescent psychiatry with a particular focus on Europe. To extend upon a global synopsis published in 2015 [1] and overviews of other specific regions (e.g., Far East [2], Europe, [3]), we have invited experts to provide a detailed overview of the training of child and adolescent psychiatrists in their countries/regions. Based on the papers in this focused issue ‘The European and global perspective on training in child and adolescent psychiatry’ [4–12], previous work and the experience and opinions of the authors, in this editorial we aim to provide an overview of the competencies already in our training that we may want to safeguard for the future and consider new competencies and skills it may be wise to include. As CAP training is firmly embedded in local care systems and society, our aim is not to provide a one-size fits all approach, but rather to provide readers with good examples around the world that can be built upon in their efforts to equip training programs to provide the trainees with a solid foundation for their future work.

Training young doctors to specialise as child and adolescent psychiatrists requires adaptation and developing greater depth in understanding the requirements for “professionalism” and “ethical practice”. In the UK all doctors are expected to follow the five tenets of the General Medical Council [13] (Ref): (i) make the care of your patient your first concern; (ii) be competent and keep your professional knowledge and skills up to date; (iii) take prompt action if you think patient safety is being compromised; (iv) establish and maintain good partnerships with your patients and colleagues; (v) maintain trust in you and the profession by being open, honest and acting with integrity. These tenets need to be understood in CAP in the context of our patients who are rapidly developing individuals in a dependent context. Their vulnerability needs to be understood and protected while also respecting their increasing autonomy as we aim to help them to develop into adults who will flourish or at least manage despite the difficulties that have brought them to our attention. Physicians must demonstrate a commitment to the principles and practice of diversity, consent and confidentiality. The very nature of work entails managing difficult and problematic relationships between patients and their carers where conflict is common. Hence, personal attributes of tact and sensitivity, and skills in negotiation/mediation are critical to our professional role. In this regard, patient and carer-centered practice, self-awareness of one’s own psychological processes and the team approach to patient care should be cornerstones of the core curriculum of CAP.

So what are the skills that a child and adolescent psychiatrist needs as we go forward? What challenges lie ahead? How do we train for these?

## Competencies related to our medical background

### Child and adolescent psychiatrists as medical doctors

Having a specialist medical voice in the assessment and treatment of children and young people with mental health difficulties and in contributing to the public health perspective of such services remains important, for several reasons. First, there are many situations where there is a strong biological basis or contribution to mental health difficulties. Second, there are often biological consequences of mental health difficulties that are best understood and addressed by a physician with skill sets, in both medicine and psychopathology. Third, the biology and psychopathology interact frequently in intricate ways and against a developmental background. They have to be considered together in an integrative diagnostic and therapeutic approach to achieve optimal care.

To fulfill child and adolescent mental health needs in the future as medical specialists, we will have to innovate to develop our assessment procedures and interventions and importantly to be leaders in the planning of effective, efficient and equitable systems of care for children and young people. In addition to emerging knowledge, there are huge gains to be achieved by implementing what we already know in terms of epidemiological need, effective care systems and currently available clinical approaches.

### Promotion of a bio-psycho-social approach

We need to promote this in collaboration with our patients and their families. A solid understanding of bio-medical knowledge of the body and mind and their complex interactions is essential for our work. Sound medical skills (e.g., history taking) and attitudes (e.g., empathy) are the foundation of every physician. We should continue to support good, current examples of medical training that focus on medical students’ needs. For example, these will include problem-based learning, early contact to patients and teaching in small groups to foster doctors that can then co-focus with their patients’ on the their needs for appropriate treatment and care.

### Skills related to psychopharmacological treatment

Drug treatments are often required to help the patient (and caregivers) to better control symptoms. Trainees must gain broad skills in both general psychopharmacology and in disorder-specific drug treatments including the appropriate and safe use of currently available pharmacological agents. To achieve this aim, trainees must understand the academic evidence base for such treatments, be able to adequately interpret randomized clinical trials and meta-analyses and apply this to individual cases. Importantly, clinical trials are usually based on study durations of a few weeks only resulting in a knowledge gap with respect to the effectiveness of long-term treatments. Patients entering clinical trials potentially bear little resemblance to the patients seen routinely. The inherent risks of over-prescribing single or multiple medications must be explicitly taught. The legal and ethical implications of drug treatments also require extensive training. Trainees must be able to critically assess the safety of drugs, particularly because the brains of our patients are still developing very actively. They may be dependent on drug treatment for prolonged time periods.

The recent biomedical advances in our understanding of the function of the brain will hopefully renew the interest of potential research and development funders, including government, industry and philanthropic, in the treatment of mental disorders. We need to teach skills to allow collaborations between partners for treatment development and potentially prevention of neurodevelopmental and other mental disorders with an early onset; at the same time trainees should learn to critically reflect the interests of partners as compared to our own to avoid conflicts of interest that impact our prescription patterns.

### Integration of novel biomedical findings into practice

We are witnessing an unprecedented increase in research and a burgeoning of empirical studies about child development, psychopathology and treatments. Research has extended into imaging, high resolution electrophysiology, molecular genetic/genomics and other highly technical neurobiological venues. A child and adolescent psychiatrist requires training to obtain a solid grasp of these fields and their clinical implications. In addition, a trainee needs guidance on how to stay informed with unbiased information and on how to be able to judge the potential impact of future developments for clinical work.

The recent molecular genetic advances in the elucidation of rare monogenic forms of mental and behavioral disorders and of polygenes and copy number variation in common mental disorders will have an increasing impact on future assessments and treatments. This development may represent just the beginning of a more biologically driven approach (proteomics, metabolomics, epigenetics) to diagnostics, which will subsequently allow for stratified treatments. Genome wide association studies have repeatedly pointed to genetic overlaps between somatic and mental disorders. These recent developments raise the possibility of overcoming the body–mind dualism allowing us to reduce the stigmatization associated with mental illness. Training will need to accommodate these findings if child and adolescent psychiatrists are to play a role in the assessment including provision of information to patients and their families and promotion of stratified treatment. Because these findings may have implications at the societal level, we need child and adolescent psychiatrists with the scientific background and authority to provide professional and ethical guidance.

### Promotion of evidence-based care

Trainees should be competent at the academic principles underlying the generation of guidelines including systematic literature search, meta-analyses, different levels of evidence and involvement of different specialists and interest groups; they need to be taught how to critically assess novel findings.

### Promotion of translational research and engagement of young clinician-scientists

Training should provide an opportunity to allow the trainee to discover his or her interest in research. In a recent editorial, young clinician-scientists of the ESCAP Research Academy pointed to the importance of further developing and supporting dual training (i.e., MD-PhD) in our field to promote a smooth translation from the lab to clinical work [14]. Even more importantly, child and adolescent psychiatry needs medical trainees with a strong interest in science [14]. Clinician-scientist careers should be systematically promoted and flexibly implemented. This refers to the amount of time spent in each field as we will need 50/50 clinician–scientists as well as clinicians with research interest and scientists with some continuing access to patients. Also, the timing of training in clinical work and scientific research should be flexibly adapted to the individual’s and the institution’s preferences and possibilities. Dual careers are highly relevant for the attainment of academic positions, which are key for promotion of research and teaching, as well as for effective lobbying. Research within child and adolescent psychiatry is pivotal for our medium and long-term survival.

## Competencies related to psychotherapy

### Integration of medical treatments and psychotherapy

It is absolutely clear that for many complex disorders psychotherapeutic treatments of one form or another, delivered individually, with families or groups, is an extremely important component of a multifocal treatment. Overall, the advances in psychotherapy have been considerable, but limitations have also become clearer especially as an isolated form of treatment for moderate and severe or complex disorders.

Many psychologists and other professionals are very proficient in the delivery of psychotherapeutic interventions. We agree that if a psychotherapeutic intervention is the sole mode of treatment required for an individual patient, the treatment and case management can be provided by another professional. It is however, crucial that a full formulation that goes beyond understanding situations solely in psychological terms and that misses biological elements, is carried out in making treatment decisions. This has implications for training child and adolescent psychiatrists and their child and adolescent mental health colleagues. We acknowledge the substantial national differences in mental health care provision and training programs should ensure acquisition of competencies required to function within the policies and funding of the respective national system. Collaborative training across disciplines should ensure that the role of the child and adolescent psychiatrist is clear to this situation. Where the recommended psychotherapeutic intervention fails to show improvement or is associated with deterioration, the child and adolescent psychiatrist should be consulted and the case should be re-evaluated. The particular CAP skills lies in their ability to coordinate and contribute to the holistic biopsychosocial treatments of children and adolescents with moderate or severe and complex disorders that are insufficiently treated with a psychotherapeutic intervention alone. To fulfil this, CAPs need to have a solid understanding and experience of psychotherapeutic interventions that can be delivered locally.

Child and adolescent psychiatrists must be able (a) to determine the relative balance of psychotherapeutic treatments with biomedical or other contextual interventions, (b) to contribute to decisions determining the most effective type of a psychotherapeutic intervention and its duration for individual cases, (c) to review the effectiveness of a psychotherapeutic treatment and (d) to work with colleagues to deploy such resources within multidisciplinary teams. Our training should focus on our capacity to address these core competencies.

Clearly, child and adolescent psychiatrists should be trained to understand and collaborate with psychotherapists when consulting to others in their assessments and interventions; also, in complex cases when asked to consult or take over a case by colleagues, child and adolescent psychiatrists have to have sufficient psychotherapeutic skills to offer a mixed treatment package. We should also be at the forefront of developments at the interface between biomedicine and psychotherapeutic interventions. We need to integrate new knowledge into psychoeducation and psychotherapy. In addition to the emergence of novel treatments we should also enable trainees to successfully implement those evidence-based treatments already available. Finally, psychotherapeutic skills are useful to minimize anxiety of patients, caregivers and colleagues in a multi-disciplinary team setting and sometimes across agencies.

In conclusion, we regard it as important that child and adolescent psychiatrists are trained to a certain level of psychotherapeutic skills to be able to treat patients and families within a multifocal treatment approach including psychotherapeutic interventions, to allow collaboration with other psychotherapists, and to provide clinical leadership. As such, psychotherapy must definitely continue to figure prominently in the curricula of CAPs. Training should have a strong focus on developing the competencies of young CAPs to that take a lead role integrating both biomedical and psychological knowledge. We also suggest that some of this will best be realized by some joint training across disciplines.

### Integration of digital revolution, artificial intelligence, e-mental health

The digital revolution in combination with artificial intelligence asks the question as to the future and type of all treatments including psychotherapy. To what extent will it need to be delivered face to face? The need for training in child and adolescent telepsychiatry (e.g., real-time bidirectional videoconferencing) has become evident in specific regions in Europe and overseas to deliver specialized mental health care to remote communities, where mental health services are grossly scarce. This way of delivering service, using different models of care, requires new skills, but also evidence-based research and development of best practice recommendations (addressing legal and ethical issues). E-mental health and virtual reality are increasingly being utilized for therapeutic purposes. Currently, companies are investing heavily to further develop emotion recognition technologies. Online treatment programs and bots based on artificial intelligence (AI) will have a strong impact requiring attention and careful scrutiny. Perhaps initial psychotherapy interventions, or psychoeducation, may be amenable to machine-based approaches. Offers will encompass a whole bouquet of self-administered online programs of diagnostics, psychoeducation and treatment tutorials to blended approaches with online or personal assistance/motivation up to full-fledged face-to-face psychotherapy. Because trainers frequently have little knowledge and experience of these recent developments, we need to ensure that trainees receive the information enabling them to firmly establish the link and make use of the digital revolution and AI, carefully assessing its evidence-base. It is important our field contributes to the practical implications for treatment and service delivery and that we remain abreast with developments.

## Competencies related to multi-professionalism, networking and the future role of child and adolescent psychiatrists in provision of mental health care services

Child and adolescent psychiatrists need to be experienced in working in multi-professional teams. Their training must capture this essential feature. Our training should allow us to achieve the prerequisites for leadership and management of large and multidisciplinary teams who focus on children and adolescents with complex mental health problems. The development throughout training of curiosity and the willingness to learn from others’ perspectives, as well the ability to share knowledge and skills are crucial in building fruitful co-operations.

### Taking roles in a stepped care system

At the level of national mental health care programs the delineation of increasingly sophisticated and cost effective tier levels of mental health care is ongoing. We need to train child and adolescent psychiatrists to understand their roles in such a stepped care system. These competencies need to be carefully thought through and incorporated into training programs. Relevant issues include (i) the role of child and adolescent psychiatrists in direct clinical and secondary consultation to other professionals in a stepped care system, (ii) clinical leadership in some specialist services, (iii) balancing patient-centered care with population-based care including the sharing of resources, (iv) training and mentorship to improve the capacity of other professionals, and (v) health care system management skills. Each of these roles needs further specification of the competencies, for instance to decide what should be included in training of consultation skills (e.g., written and summary skills for other professionals), the capacity to review treatments, training and overseeing others and leadership in clinical systems. We need to enable future child and adolescent psychiatrists to reach out to government and other professionals/institutions in the field to improve the overall management of youths with mental disorders; to be capable advocates for policy and to learn the skills of collaborative approaches with experts by experience (often ex-patients), parents and carers as well as with other professionals. We need to more systematically provide child and adolescent psychiatrist with the relevant skills including public health analysis and advocacy and developing patient friendly care systems. Many child and adolescent psychiatrists will not be involved in this type of work on a daily basis but many find themselves in this situation from time to time in their careers.

### Prevention and early intervention

Societal factors such as poverty, poor living conditions, abuse and many other factors increase the prevalence of mental health disorders. They have a marked effect on the economies of our countries, let alone the distress and impairment caused. Much is said about prevention and early intervention; while evidence exists for particular intervention, good examples of successful broad upscaling of programs are limited. Child and adolescent psychiatrists need to understand the differences between mental health promotion, prevention and various levels of early intervention. All child and adolescent psychiatrists should receive some training to support health promotion, prevention, resilience and early intervention in their communities.

### Assure transition to adult psychiatry

Transitioning to adult psychiatry services represents a critical time for young people with mental health disorders. Continuity of psychopathology does not match the current structures of mental health care systems; the gap between adolescent and adult services is a major barrier that does not serve our patients well. Transitioning is particularly needed for neurodevelopmental disorders and early onset psychoses but also for the large proportion of adult mental health disorders that commence in adolescence. The introduction of a shared mandatory transition training for CAPs and adult psychiatry trainees that follows a developmental approach would greatly assist. Further, identification of the optimal amount of child and adolescent training in adult psychiatry and vice versa should be urgently considered, as well as a harmonization of curricula, which in Europe appear to be very heterogeneous [5, 15, 16].

### Teaching and learning skills

Educating and sharing our knowledge is important for our young patients and their parents as well as with other adults who contribute to their welfare including teachers, nurses and our other medical colleagues. Further integration of psychiatric, psychosomatic and psychological contents into medical training is urgently warranted to support a holistic bio-psycho-social approach early in the career of every physician. Such efforts will boost the collaboration between medical specialists and ensure that every specialist has a solid knowledge of mental functioning and mental disorders and how to address these in his/her interaction with patients. Physicians need to be trained to decide when the input of a child and adolescent psychiatrist is required to avoid delays in assessment and treatment of an underlying or contributing mental disorder.

Through promotion of the bio-psycho-social approach within our medical schools we generate interest, understanding and enthusiasm among medical students for our field. The future of child and adolescent psychiatry critically depends on a sufficiently high influx of capable physicians with the desire to train and continuously shape our field. We need to ensure child and adolescent psychiatry is a highly rewarding and attractive field and expose medical students and junior doctors to our exciting profession. Interest for our field will benefit from the substantial advances in the neurosciences which remind us that symptoms of the mind, like other medical symptoms, originate from an organ (i.e., the brain). These advances in neuroscience deserve credit for placing the brain central in recent developments in child and adolescent psychiatry. They require CAPs to have sufficient neuroscience training to understand advances in the field and the teaching skills to interpret these findings for others who live and work with our patients.

### Training to manage twenty-first century risks and vulnerabilities

Current global challenges may impact the mental health of children and young people. Prominent examples include migration, wars, and terrorist attacks which all have obvious implications for the mental health of children. Fridays for Future has developed as a youth based movement; the fears related to climate change but also the slow progress with respect to environmental changes to reduce carbon dioxide emissions is already beginning taking its toll on vulnerable adolescents. It seems likely that climate change and its impacts will affect tomorrow’s mental disorders and require child and adolescent psychiatrists to respond to an evolving challenge. There are other risks and opportunities in the ever changing social media. Gender dysphoria is becoming more prominent. In summary, we need to prepare trainees in our field for these challenges, to come up with innovative strategies to help young people who develop serious disorders associated with these challenges.

Recent European history has revealed that migration is a world-wide issue that does not stop at our borders. It is hard to envisage that migration will NOT continue over the next decades. It has become evident that it is by no means easy to provide effective treatment due to for instance language or cultural barriers. We need to train child and adolescent psychiatrists to provide culturally sensitive mental health care to immigrant and refugee children and their families, who often face or have survived the additional stressors of exposure to the traumas of war, torture, persecution, or natural disasters, the process of immigration, and changes in family structure and dynamics forced by these events. Unaccompanied minors are a particularly vulnerable group of refugees in need of special protection and support. Contextual understanding of psychosocial, cultural challenges they face are relevant to clinical issues and psychopathology they might exhibit. Teaching our trainees to be sensitive, to and have the skills to address these are imperative in working with them directly or in providing consultation to a system of care for them. Trainees need to learn the skills of mental health in the context of culturally-regulated child-rearing practices, expected roles, patterns of communication, acceptable behavior, and coping mechanisms, when interpreting the meanings of behavior in order to avoid misdiagnosis and misguidance. This is a challenge to us all to learn these skills to an effective level.

## Outlook

In conclusion, child and adolescent psychiatrists need the competencies to deal with complex disorders that have their origins in the biological, psychological, systemic, contextual and cultural domains. Our training programs need to remain anchored in medicine; a trainee should not shy away from patients with (comorbid) somatic disorders, but instead be keen to examine the potential link to the mental disorder(s) the patient presents with. This requires knowledge and interest in researching and understanding the complex interface between the somatic and psychological and the implications for the care of the patient.

We hope that we have illustrated that a career as a CAP is an exciting and stimulating choice for young physicians by pointing out the complex challenges and broad skill set required that lie ahead to be a competent child and adolescent psychiatrist. Young individuals embarking in their specialization will rightly demand to know how they are to obtain all the skills deemed necessary in this editorial. They will also rightly ask us how to manage new skills on top of all the knowledge that we considered basic but which nevertheless constitutes the everyday clinical knowledge required for our routine work. And they may point out that just adding more is not helpful if we do not at the same time understand what to disregard as no longer essential for the future. It was our intention to open the discussion on our future identity and role as child and adolescent psychiatrists within child and adolescent mental health care, thus stimulating a revision of existent curricula as we move into the future. We realize that within this large framework every child and adolescent will find his or her place based on individual interests and strengths. Let us jointly tackle the issues up ahead with excitement and curiosity!
